# Evaluation of an Artificial Intelligence Conversational Chatbot to Enhance HIV Preexposure Prophylaxis Uptake: Development and Usability Internal Testing

**DOI:** 10.2196/79671

**Published:** 2026-02-03

**Authors:** Jun Tao, Ellie Pavlick, Amaris Grondin, Josue D Bustamante, Harrison Martin, Hannah Parent, Natalie Fenn, Alexi Almonte, Amanda Maguire-Wilkerson, Mofan Gu, Jack Rusley, Bryce K Perler, Tyler Wray, Amy S Nunn, Philip A Chan

**Affiliations:** 1 Department of Medicine, The Warren Alpert Medical School of Brown University, Room 130, 11 4th Street, Providence, RI, 02906, United States, 1 401-863-1000; 2 Department of Computer Science, Brown University, Providence, RI, United States; 3 School of Electrical Engineering and Computer Science, College of Engineering, Oregon State University, Corvallis, OR, United States; 4 Department of Psychiatry and Human Behavior, The Warren Alpert Medical School of Brown University, Providence, RI, United States; 5 Division of Infectious Diseases, The Miriam Hospital, Providence, RI, United States; 6 Department of Health Services, Policy, and Practice, Brown University School of Public Health, Providence, RI, United States; 7 Department of Pediatrics, Division of Adolescent Medicine, The Warren Alpert Medical School of Brown University, Providence, RI, United States; 8 Department of Behavioral and Social Sciences, Brown University School of Public Health, Providence, RI, United States; 9 Open Door Health, Rhode Island Public Health Institute, Providence, RI, United States

**Keywords:** artificial intelligence, counseling, HIV infections, motivational interviewing, multiagent framework, preexposure prophylaxis

## Abstract

**Background:**

The HIV epidemic in the United States disproportionately impacts gay, bisexual, and other men who have sex with men (MSM). Despite the effectiveness of HIV preexposure prophylaxis (PrEP) in preventing HIV acquisition, uptake among MSM remains suboptimal. Motivational interviewing (MI) has demonstrated efficacy at increasing PrEP uptake among MSM but is resource-intensive, limiting scalability. The use of artificial intelligence, particularly large language models with conversational agents (ie, “chatbots”) such as ChatGPT, may offer a scalable approach to delivering MI-based counseling for PrEP and HIV prevention.

**Objective:**

This internal usability testing aimed to evaluate the development of an artificial intelligence–based chatbot, including its ability to provide MI-aligned education about PrEP and HIV prevention and potential to support PrEP uptake.

**Methods:**

The Chatbot for HIV Prevention and Action (CHIA) was built on a GPT-4o base model embedded with a validated knowledge database on HIV and PrEP in English and Spanish. The CHIA was fine-tuned through training on a large MI dataset and prompt engineering. The use of the AutoGen multiagent framework enabled the CHIA to integrate 2 agents, the PrEP Counselor Agent and the Assistant Agent, which specialized in providing MI-based counseling and handling function calls (eg, assessment of HIV risk), respectively. During internal testing from March 10-April 28, 2025, we systematically evaluated the CHIA’s performance in English and Spanish using a set of 5-point Likert scales to measure accuracy, conciseness, up-to-dateness, trustworthiness, and alignment with aspects of the MI spirit (eg, collaboration, autonomy support) and MI-consistent behaviors (eg, affirmation, open-ended questions). Descriptive statistics and mixed linear regression were used to analyze the data.

**Results:**

A total of 296 responses, including 145 English responses and 151 Spanish responses, were collected during the internal testing period. Overall, the CHIA demonstrated strong performance across both languages, receiving the highest combined scores in the general response quality metrics including up-to-dateness (mean 4.6, SD 0.8), trustworthiness (mean 4.5, SD 0.9), accuracy (mean 4.4, SD 0.9), and conciseness (mean 4.2, SD 1.1). The CHIA generally received higher combined scores for metrics that assessed alignment with the MI spirit (ie, empathy, evocation, autonomy support, and collaboration) and lower combined scores for MI-consistent behaviors (ie, affirmation, open-ended questions, and reflections). Spanish responses had significantly lower mean scores than English responses across nearly all MI-based metrics.

**Conclusions:**

Our internal usability testing highlights the potential of the CHIA as a viable tool for delivering MI-aligned counseling in English and Spanish to promote HIV prevention and support PrEP uptake, though its Spanish language performance requires further improvement.

## Introduction

In the United States, HIV continues to be a significant cause of morbidity and mortality, disproportionately affecting groups including gay, bisexual, and other men who have sex with men (MSM). In 2022, MSM comprised 67% of new HIV diagnoses in the United States [1]. Hispanic or Latino and Black or African American individuals accounted for 39% and 35% of these new diagnoses, respectively [1]. HIV preexposure prophylaxis (PrEP) is highly effective at preventing HIV among populations at an increased risk of infection, including MSM [2-4]. However, PrEP uptake among MSM, and particularly Hispanic or Latino and Black or African American MSM, remains suboptimal due to inequitable access to health care and stigma [5-7].

 Motivational interviewing (MI) is an evidence-based, patient-centered approach to healthy behavior change that has demonstrated efficacy in facilitating PrEP uptake among MSM [8-10]. In our previous work, we demonstrated that a brief MI-based intervention improved PrEP uptake among MSM in a clinical setting [9]. However, implementing MI in practice requires a significant investment in time and resources for provider training and intervention delivery. Research suggests that provider-led MI counseling usually requires 2-5 sessions (30‐60 min per session) to produce measurable behavior change, with several studies reporting substantially higher effectiveness (>80%) when more than 5 sessions are delivered [11-13]. The associated clinician time and continuity demands make sustained MI delivery difficult to scale, particularly during brief clinical visits [11-14]. Time demands apply not only to patient encounters but also to provider training, which typically involves several hours of didactic instruction and coaching [13]. Artificial intelligence (AI)—specifically large language models (LLMs) with conversational agents (“chatbots”) such as ChatGPT—has shown promise in overcoming these challenges [15-17]. Preliminary research supports AI-based chatbots’ ability to employ MI techniques in promoting healthy behavior change, including smoking cessation and decreased substance use [17-21]. A chatbot designed to use MI principles can be made available 24/7, requiring only minimal human effort for periodic supervision and quality assurance. Drawing on empirical session-length data and standard labor-cost benchmarks, it suggests that such MI-aligned chatbots could substantially improve the scalability of MI delivery.

In the context of HIV prevention, researchers have noted many uses for AI-based chatbots [22-24]. Limited evidence suggests that chatbots may help facilitate the uptake of HIV testing and PrEP among populations at increased risk of HIV, including MSM [22,23,25]. Chatbots have also shown the potential to provide personalized counseling on sensitive health topics including HIV prevention [22]. Although AI-based chatbots hold significant promise for HIV prevention efforts, concerns exist regarding their ability to provide accurate medical information, stay up-to-date on current clinical recommendations, and demonstrate cultural competence [22,26]. Studies have highlighted issues such as hallucinations (incorrect or misleading information) and the potential for perpetuating biases, which can undermine trust and effectiveness [22]. Low engagement with AI-based chatbots for health promotion has also been documented in the literature, presenting challenges with delivering effective interventions via this modality [27,28]. Additionally, no studies, to our knowledge, have evaluated the use of MI by an AI-based chatbot for HIV prevention.

In this usability testing, we developed and conducted an internal evaluation of an AI-based chatbot (Chatbot for HIV Prevention and Action [CHIA]) that harnesses MI to provide personalized counseling for PrEP. To address the limitations noted above and improve the CHIA’s performance, we integrated three complementary components: (1) a retrieval-augmented generation pipeline constrained to a curated, validated knowledge base to reduce unsupported statements; (2) MI alignment via supervised fine-tuning on annotated MI transcripts coupled with preference-based tuning using expert-selected responses; and (3) personalization through a structured HIV risk assessment and the transtheoretical model (TTM) to tailor counseling to the user’s stage of change [29,30]. We present the results of an internal evaluation of the CHIA, assessing the chatbot’s alignment with MI principles, factual accuracy, and its ability to deliver appropriate counseling for HIV prevention and PrEP. This internal testing serves as a cornerstone for future real-world implementation and evaluation of the CHIA’s performance among individuals at an increased risk of HIV.

## Methods

### Overview of Chatbot Design

Generative pre-trained transformers are LLMs that use deep learning to generate human-like text based on natural language input [31]. GPT-4o, released by OpenAI in May 2024, is a multimodal LLM capable of processing both text and images with improved efficiency and performance compared to earlier versions [32]. We developed the CHIA using GPT-4o to deliver MI-informed counseling aimed at improving HIV prevention outcomes, particularly the uptake of PrEP.

The CHIA consists of two main components: (1) a fine-tuned, customized LLM embedded with a validated knowledge database and (2) multiple specialized agents and functions to meet users’ needs. Built on a GPT-4o base model, the CHIA detects language inputs automatically and has been fine-tuned using a large MI dataset with the goal of training it to produce empathetic responses and avoid biases [33,34]. GPT-4o was selected because it offers superior conversational quality, multilingual capability, and reduced risk of generating errors compared to smaller open-source models. These features are critical for building trust with users discussing sensitive health topics. To ensure accurate and up-to-date information on HIV and PrEP, the CHIA integrates the latest validated data on these topics, reviewed by a team of physicians and researchers, in English and Spanish, with monthly updates.

**Figure 1.**
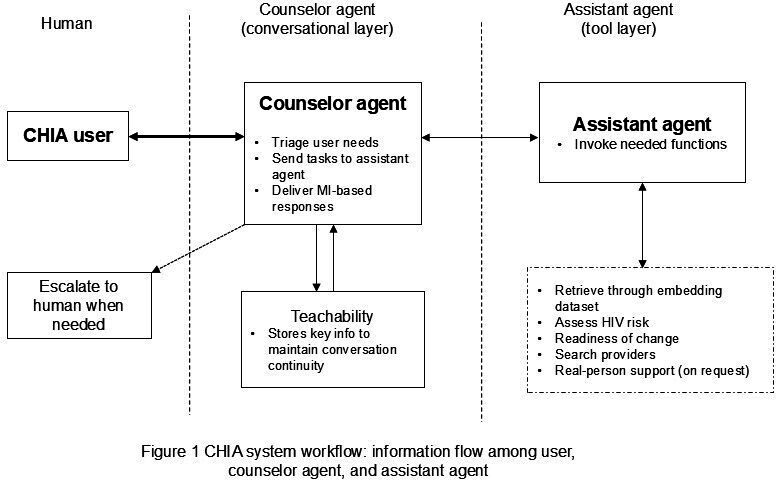
Chatbot for HIV Prevention and Action (CHIA) architecture: information flow among User, Counselor Agent, and Assistant Agent. MI: motivational interviewing.

The CHIA’s architecture integrates 2 specialized agents (Figure 1): a user-facing PrEP Counselor agent and a tool-executing Assistant agent using AutoGen [35]—an open-source multiagent framework developed by Microsoft. The Counselor conducts the entire conversation using MI, triages user needs, and delegates tasks to the Assistant when external information is required. The Assistant then invokes specific functions—knowledge retrieval from an embedding-backed knowledge base, HIV risk or readiness assessment, PrEP provider search and referral info, reminders or links, and initiating human support on request—and returns results to the Counselor. The Counselor interprets these outputs through MI principles and delivers the response to the user. To maintain continuity across visits, the Counselor agent is capable of using *Teachability* to store key information to ensure the continuity of conversation (eg, identity token, risk or concerns, prior plan or notes). This design enables the CHIA to deliver personalized responses that mimic human counseling. Advanced prompt engineering keeps interactions dynamic and contextually relevant [36]. The CHIA is secured through a login page and deployed on Amazon Web Services (AWS) with the Supabase software (version 1.25; Supabase, Inc.) for backend management, ensuring robust and private data handling [37,38].

### Embedded HIV/PrEP Dataset Development and Validation

To enhance chatbot accuracy and reliability, embedding and retrieval-based techniques have been employed to minimize misinformation by ensuring responses are grounded in validated sources [39]. Assessment retrieval methods, guided by established frameworks, such as the retrieval-augmented generation (RAG) playground framework and the automated RAG evaluation system [40-42], allow for systematic evaluation of chatbot performance in delivering evidence-based, contextually relevant health information. To develop a robust embedded dataset specifically for the CHIA, our study team, composed of 4 research assistants under the guidance of 2 physicians and 1 principal investigator with decades of research experience in HIV prevention and treatment, systematically curated and validated key information. We compiled the most frequently asked questions about HIV and PrEP, which included topics such as basic knowledge, effectiveness, side effects, formulations, insurance coverage, financial assistance, and cultural considerations. A Spanish-fluent research assistant translated the dataset to ensure accessibility for Spanish-speaking users. Each data entry was structured into a standardized format, categorizing content by topic, question, and answer to optimize retrieval efficiency. All information underwent expert validation for medical accuracy and clarity. These processes prevent the spread of misinformation or erroneous recommendations (“hallucinations”), which ChatGPT cannot guarantee. The embedding database is updated monthly and as needed when users ask questions outside the existing dataset under expert supervision. In such cases, the system flags the query, alerting the development team to review, validate, and integrate new information. This dynamic updating process helps to ensure that the CHIA remains accurate, relevant, and responsive to evolving needs of the community.

### MI Alignment via Implementation of Preference Fine-Tuning Techniques

To enhance the CHIA’s ability to deliver MI-based counseling, we utilized preference fine-tuning techniques [43]. This was done because open-source models would have been insufficient for MI fine-tuning due to their lack of multilingual support, higher hallucination rates, and insufficient token context. We processed a large, publicly available MI dataset downloaded from GitHub to facilitate diversity in linguistic styles and conversational structures [33,34]. The dataset contained 2000 dialogs, half of which were from publicly available conversations between potential clients and licensed counselors on CounselChat—an online platform for mental health support—and the other half from exchanges between users and peer supporters on Reddit subforums related to emotional distress [33,34]. All dialogs in the dataset were annotated by trained counselors with labels adapted from the Motivational Interviewing Treatment Integrity Code 2.0 and 4.2.1, a widely used method for evaluating how well clinicians perform MI [33,34,44-46]. Our team preprocessed the raw text from the dataset, tokenized it (ie, broke down into smaller units) [47], and then converted this to a vector database. This processed vector database was employed to fine-tune the GPT-4o base model, enabling the CHIA to generate responses informed by MI. We used the Direct Preference Optimization algorithm to refine response quality [43]. A preference fine-tuning JSON file was constructed that ranked certain responses as preferable over others based on their alignment with MI-consistent behaviors such as open-ended questioning and reflective listening. This iterative training process was designed to enhance the CHIA’s ability to prioritize MI-aligned responses while maintaining coherence and engagement. By the end of this process, we developed a specialized ChatGPT-4o model for MI-based counseling, serving as the foundation for the CHIA to deliver personalized, empathetic, and structured conversations that support PrEP uptake and public health interventions.

### HIV Risk Assessment and Readiness for Change Functions

To enhance the CHIA’s ability to provide personalized guidance, we integrated functions to assess individual HIV risk and readiness for behavior change. HIV risk assessment was based on the HIV Incidence Risk Index for MSM [48], following Centers for Disease Control and Prevention guidelines for PrEP eligibility [49]. A focus on MSM was chosen due to the disproportionate number of new HIV diagnoses occurring among this population in the United States [1]. Additionally, the CHIA’s development incorporated the TTM through the use of the Contemplation Ladder, a validated tool that allowed individuals to self-assess their readiness for PrEP uptake on a scale from 0 to 10 [29,50]. These assessments were embedded into the CHIA’s conversational interface, enabling real-time evaluation, as users engaged with relevant questions. Based on these assessments, the CHIA tailored its responses to align with each user’s unique risk profile and stage of readiness for change, providing targeted, motivational, and evidence-based guidance to support PrEP uptake.

### Linkage to PrEP Care and Referral Function

To facilitate access to PrEP, we developed a function that searched the “PrEP Locator” website, a national database of PrEP providers in the United States, using user-provided ZIP codes [51]. This function was integrated into the CHIA to generate a list of PrEP providers within a 30-mile radius, offering users convenient options for selecting nearby clinics. Additionally, a separate function was implemented to enable the CHIA to engage users with follow-up questions to assess clinic preferences, determine if they wished to be contacted by a provider for an initial appointment, and identify potential barriers to care. An AI-generated and encrypted email was then sent to the selected clinic with a referral and relevant contact information. Study staff coordinated with the clinic to ensure appointments were scheduled efficiently. Furthermore, the CHIA inquired whether users preferred to connect with a real person for assistance in accessing PrEP care. If requested, study staff were notified and contacted the individual within 1 business day.

### User Interface and Data Security

**Figure 2.**
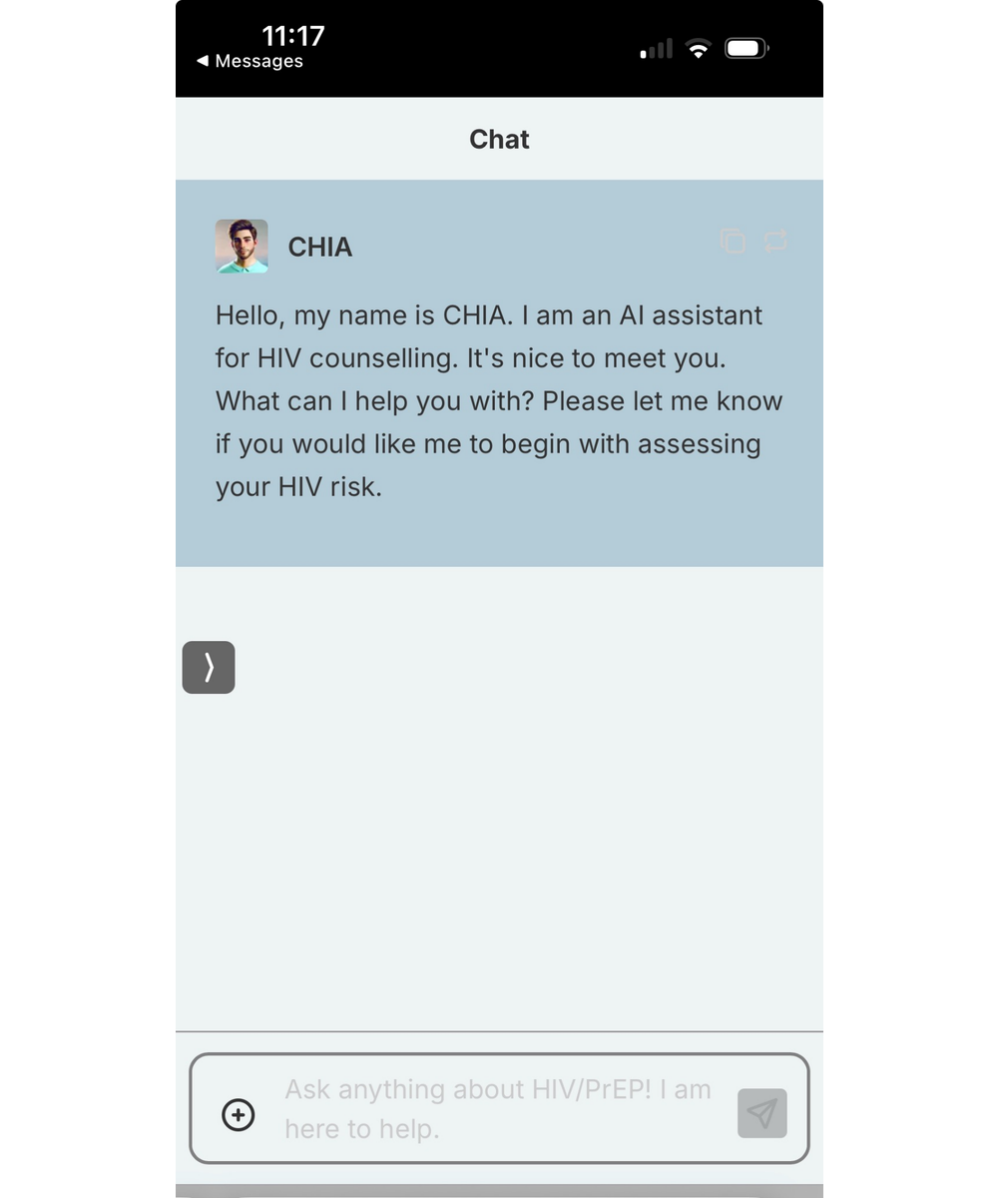
The user interface of the Chatbot for HIV Prevention and Action (CHIA).

The CHIA user interface was designed to provide a streamlined, secure, and personalized experience (Figure 2). Users could log in with an existing account or create a new one, with 2-factor authentication required for each login to ensure data protection and confidentiality. The login system adhered to high cybersecurity standards to safeguard user information. Once logged in, users accessed a clean, intuitive interface with clear navigation options and a responsive chat window to facilitate seamless interactions.

To facilitate secure data management, the CHIA was deployed through AWS, leveraging its cloud-based infrastructure for reliable and Health Insurance Portability and Accountability Act (HIPAA)–compliant storage [38]. AWS encryption protocols protected user data, ensuring confidentiality and integrity [38]. Additionally, Supabase was used to host the back-end data, providing an efficient database solution for managing user interactions [37]. This integration of AWS for deployment and Supabase for back-end data management enabled the CHIA to maintain robust security measures while promoting a smooth user experience. The secure, user-friendly interface aimed to encourage sustained engagement and support meaningful interactions.

### Procedures

During internal testing from March 10 to April 18, 2025, 4 research staff members systematically evaluated the CHIA’s performance in both English and Spanish. Each researcher acted as a potential user and interacted with the chatbot over multiple sessions. Two research staff members interacted with the chatbot in English, and 2 staff members fluent in Spanish interacted with the chatbot in Spanish. Researchers then exchanged transcripts and rated each response on a set of 11 metrics covering general response quality and alignment with MI using a 5-point Likert scale. Likert scores were used primarily to enable relative comparisons across languages and scenarios, rather than to assess performance against an external benchmark, as no standardized threshold currently exists for interpreting Likert-based ratings of motivational interviewing alignment in chatbot evaluations. The research team met prior to the review process to ensure familiarity with the operational rating scale and consistency in scoring across raters. After interacting with the chatbot, the team met to discuss conversation-level findings and the performance of the HIV risk assessment, referral, and readiness for change functions.

### Evaluation Metrics for Retrieving Information From Embedded Dataset

The CHIA’s RAG functionality was evaluated at the session level using 7 key metrics—groundedness, medical accuracy, completeness, no fabrication, appropriate tone, safety, and reasoning—that were scored on a 5-point Likert scale via the Automated RAG Evaluation System [42,52]. Groundedness assessed if responses were based on retrieval sources, and medical accuracy sought to ensure alignment with validated health information. Completeness measured whether the retrieved content provided sufficient information. No fabrication verified that responses did not introduce false or misleading details (sometimes referred to as “hallucinations”) by comparing each RAG output against the ground-truth dataset to confirm retrieval only from the embedded validated knowledge base. Appropriate tone was evaluated if responses were professional and empathetic, and safety was assessed whether the information adhered to ethical and safety principles. Finally, reasoning evaluated the chatbot’s ability to integrate and apply retrieved knowledge. Sessions with any metric below a predefined threshold were auto-flagged for human review; we logged and reviewed misinformation flags and near-miss events (eg, unsafe advice avoided or corrected) and conducted monthly audits summarizing flag rates, time-to-review, and corrective actions.

### Metrics for Comprehensive Assessment

We developed and used a structured assessment framework to evaluate the CHIA’s responses based on previous research that incorporated multiple dimensions of chatbot interaction [26,53]. First, we assessed general response quality using 4 metrics—accuracy, conciseness, up-to-dateness, and trustworthiness—that were scored on a 5-point Likert scale to ensure that responses were factually correct, concise, up-to-date, and emotionally supportive. We also developed a set of metrics inspired by the Motivational Interviewing Treatment Integrity Code to measure the alignment of the CHIA’s responses with the MI spirit and MI-consistent behaviors [44,45]. Metrics to assess alignment with the MI spirit included empathy, evocation, autonomy support, and collaboration, and those for MI-consistent behaviors included affirmation, open-ended questions, and reflections. All MI-based metrics were also scored using a 5-point Likert scale. Additionally, safety was evaluated on a pass or fail basis in which the CHIA’s use of toxic language, demonstration of bias, or violation of privacy constituted a failure. A qualitative feedback section allowed raters to document strengths, weaknesses, and suggested improvements. This comprehensive framework ensured a robust and reliable evaluation of the CHIA’s performance in multiple languages and user engagement scenarios. The detailed metrics and operational assessment protocol is included in Multimedia Appendix 1. At the conversation level, we assessed the CHIA’s performance using the same response-level metrics in addition to evaluating its overall adherence to MI techniques including employing a guiding (rather than directive) style of conversation, eliciting change talk, and managing sustain talk (ie, statements against change). Given that this analysis was limited to 4 conversations, only qualitative findings are reported in this paper. Finally, we assessed the CHIA’s memory and teachability by measuring its ability to recall information accurately and integrate key details from past interactions into current conversation. The qualitative findings of this assessment are summarized in this paper.

### Ethical Considerations

This internal testing did not involve human participants or the use of human subject data. All testing was conducted internally using simulated interactions to evaluate chatbot performance. As such, this developmental phase of the study does not meet the definition of human participants research and was determined to be exempt from IRB review by the Miriam Hospital Institutional Review Board (protocol #2312729).

### Statistical Analysis

Descriptive statistics, including the mean and SD, were calculated separately for English and Spanish responses for each metric (ie, accuracy, conciseness, up-to-dateness, trustworthiness, empathy, evocation, autonomy support, collaboration, affirmation, open-ended questions, and reflections). We fit mixed linear regression models to estimate the effect of language (Spanish vs English) on each communication metric. Because multiple observations were nested within individuals, models included random intercepts and random slopes for language at the participant level. This specification accounted for within-person correlation (ie, repeated measures from the same individual) and allowed the magnitude of the language effect to vary across individuals. Fixed effects provided the average adjusted difference between languages, while random effects decomposed variance into within- and between-person components. In addition, standardized effect sizes (Cohen *d*) were derived by dividing the adjusted language difference by the residual standard deviation, providing a measure of the practical significance of language effects across metrics. Statistical significance was assessed using 2-tailed *P* values. The significance level was set at *P*<.05. All analyses were performed using Stata (version 18; StataCorp LLC).

## Results

### Overview of Response-Level Assessment

A total of 296 responses were assessed across 11 metrics, covering general response quality (eg, accuracy, trustworthiness) as well as alignment with the aspects of the MI spirit (eg, evocation, autonomy support) and with MI-consistent behaviors (eg, affirmation, open-ended questions). This total included 145 English responses and 151 Spanish responses. All responses in English and Spanish passed the safety evaluation, indicating that the CHIA’s outputs were appropriate, ethical, and unbiased. Table 1 presents the mean and SD for all metrics at the response level, along with effect sizes reported using Cohen *d*. Table 2 displays the examples of CHIA’s responses to researcher prompts and their scores for evaluation metrics. Multimedia Appendix 2 includes all responses and scores used in the analysis for both the English and Spanish assessments.

**Table 1.** Response-level assessment for the internal testing phase of Chatbot for HIV Prevention and Action (CHIA), an artificial intelligence (AI)–based chatbot for HIV prevention.

Response-level metrics	English (n=145), mean (SD)	Spanish (n=151), mean (SD)	Total (n=296), mean (SD)	ICC^a^	Cohen *d*	*P* value
General response quality metrics						
Accuracy	4.1 (1.1)	4.6 (0.7)	4.4 (0.9)	7.65e-15	−0.55	<.001
Conciseness	4.0 (1.3)	4.5 (0.8)	4.2 (1.1)	0.120	−0.47	.07
Up-to-dateness	4.5 (1.0)	4.7 (0.7)	4.6 (0.8)	0.035	−0.24	.24
Trustworthiness	4.3 (1.0)	4.7 (0.7)	4.5 (0.9)	0.0169	−0.47	.001
MI^b^ spirit metrics						
Empathy	4.2 (1.0)	3.9 (1.2)	4.1 (1.1)	0.242	0.272	.95
Evocation	4.0 (1.2)	2.0 (1.1)	3.2 (1.5)	0.257	1.74	.24
Autonomy support	4.2 (1.1)	3.7 (1.3)	3.9 (1.2)	0.279	0.413	.84
Collaboration	4.1 (1.1)	3.7 (1.4)	3.9 (1.3)	0.357	0.313	.786
MI-consistent behaviors metrics						
Affirmation	4.2 (1.2)	2.7 (1.6)	3.5 (1.6)	0.607	1.05	.001
Open-ended questions	3.8 (1.3)	3.0 (1.9)	3.4 (1.7)	0.536	0.48	.95
Reflections	3.6 (1.3)	3.7 (1.4)	3.6 (1.3)	0.452	−0.07	.59

a ICC: intraclass correlation coefficient.

b MI: motivational interviewing.

**Table 2.** Response-level assessment examples and scores^a^.

		Evaluation metric						
Researcher prompt	CHIA^b^ response	Empathy	Evocation	Autonomy support	Collaboration	Affirmation	Open-ended questions	Reflections
I’ve tried setting timers before for medications and they don’t work.	It sounds like you’ve tried different strategies without success, and that’s okay. Sometimes it takes a bit of creativity to find what works best for you. Perhaps pairing PrEP^c^ with a daily activity you never miss, like eating breakfast, could help. Would you like to explore other ways to create a routine that feels natural for you?	5	5	5	5	5	5	5
Is there someone I can talk to about PrEP that is not a doctor or works in healthcare? I don’t trust doctors; in my experience, they’ve treated me poorly.	I’m sorry to hear about your experiences. Seeking support from someone outside of healthcare is a valid choice. Community organizations or support groups focused on sexual health might be a good option. They can offer guidance and answer questions about PrEP. How do you feel about reaching out to a local LGBTQ+^d^ center or community health organization for support?	5	5	5	5	5	5	5
My partner could also benefit from PrEP maybe. How do I talk to my partner about it?	Talking to your partner about PrEP can be an important step in taking care of your sexual health together. You might start by expressing your care and concern for both of your well-being. You could say something like, “I’ve been thinking about how we can protect each other from HIV and STIs^e^, and I came across PrEP. It’s a medication that can help reduce the risk of HIV, especially if we have sex without condoms sometimes. What do you think about looking into it together?” This approach opens up a dialogue and allows your partner to share their thoughts and feelings, making it a collaborative decision.	4	1	4	1	4	4	4

a Responses were obtained through internal testing, in which research assistants simulated potential participants from the target study sample. These responses were not collected from actual human participants within the study sample. Please see Supplemental Dataset 1 for further examples of CHIA responses, including Spanish responses.

b CHIA: Chatbot for HIV Prevention and Action.

c PrEP: preexposure prophylaxis.

d LGBTQ+: lesbian, gay, bisexual, transgender/transsexual, queer, and other minority sexual orientations and gender identities.

e STIs: sexually transmitted infections.

### English Response-Level Assessment

The CHIA performed strongly in up-to-dateness (mean 4.5, SD 1.0), trustworthiness (mean 4.3, SD 1.0), and accuracy (mean 4.1, SD 1.1). Conciseness had a slightly lower score (mean 4.0, SD 1.3) but remained generally acceptable. Metrics that assessed alignment with the MI spirit generally received higher mean scores than those for MI-consistent behaviors across English responses.

### Spanish Response-Level Assessment

Spanish responses demonstrated strong performance across several dimensions. General response quality metrics including trustworthiness (mean 4.7, SD 0.7), up-to-dateness (mean 4.7, SD 0.7), accuracy (mean 4.6, SD 0.7), and conciseness (mean 4.5, SD 0.8) were rated highly. Overall, metrics that measured alignment with the MI spirit and MI-consistent behaviors received lower combined scores.

### Combined Response-Level Assessment

The CHIA performed well across both languages, with combined scores showing strength across general response quality metrics including up-to-dateness (mean 4.6, SD 0.8), trustworthiness (mean 4.5, SD 0.9), accuracy (mean 4.4, SD 0.9), and conciseness (mean 4.2, SD 1.1). The mean scores for accuracy, conciseness, and trustworthiness were significantly higher among Spanish responses compared to English responses. Combined scores for metrics that assessed alignment with the MI spirit were generally higher than those for MI-consistent behaviors. Statistical tests indicated that Spanish responses received significantly lower mean scores than English responses across nearly all MI-based metrics.

### Conversation Level Assessment

Conversations with the CHIA were generally perceived as reliable and emotionally supportive but occasionally repetitive or overly generic. Empathy and collaboration were present but could be deepened with more emotionally attuned language and user-specific questions. The CHIA’s autonomy support was acknowledged, though one instance where the chatbot proceeded with a risk assessment against user preference indicated room for technical and conversational improvements. All reviewers emphasized reducing reliance on early referrals to health care providers and instead suggested a more user-driven flow. MI techniques (eg, change talk elicitation, sustain talk management, guiding style) were successfully implemented across English conversations, yet raters noted the need for the CHIA to ask more personalized, open-ended questions earlier in the conversation to build rapport and relevance. In the Spanish version, reviewers reported that conversations were not consistently MI-aligned, often lacking key aspects of the MI spirit such as autonomy support and MI-consistent behaviors including affirmation, open-ended questions, and reflections.

### Evaluation of the Referral, HIV Risk Assessment, and Readiness for Change Functions

For the referral function, ZIP codes from across the United States were entered. The CHIA successfully returned accurate listings of nearby PrEP clinics within a 30-mile radius. However, the chatbot occasionally failed to provide detailed information about specific clinics when requested, highlighting the need to enable the CHIA to retrieve location-specific data by accessing selected clinic websites. In contrast, the HIV risk assessment function consistently performed well across all conversations. This feature has since been refined to allow users to exit the assessment if it is accidentally triggered. Overall, both referral and risk assessment functions were functional and helpful, with minor refinements needed to optimize user experience. All research assistants tested the CHIA’s readiness for change function, which supports MI-based counseling by identifying the user’s stage of change. The function successfully prompted tailored, stage-appropriate responses to guide users toward PrEP decision-making. Overall, it enhanced the CHIA’s ability to deliver personalized, action-oriented support aligned with MI core skills in this internal pilot testing.

### Assessment of Retrieval Functionality

Performance was strong across all 7 key metrics designed to assess the CHIA’s RAG functionality (groundedness, medical accuracy, completeness, no fabrication, appropriate tone, safety, and reasoning; Table 3). Mean scores ranged from 3.7 to 4.6, with SDs between 0.5 and 1.1. Median scores for each metric were consistently high, with IQRs falling within acceptable variability (eg, median 4, IQR 3-5). These findings indicate that the CHIA’s responses were consistently accurate, grounded in reliable sources, and communicated in a safe and professional manner.

**Table 3.** Evaluation of the Chatbot for HIV Prevention and Action’s (CHIA) retrieval-augmented generation functionality.

Metrics	Mean (SD)
Groundedness	4.1 (1.1)
Medical accuracy	4.6 (0.8)
Completeness	3.7 (1.0)
No fabrication	4.6 (0.8)
Appropriate tone	4.6 (0.6)
Safety	4.7 (0.5)
Reasoning	3.9 (1.0)

### Assessment of Memory and Teachability

Preliminary tests indicated that the CHIA was able to successfully recall information from prior discussions when prompted. Additionally, following the implementation of the teachability feature, an overall reduction in repetitive information provided to the user was noted, highlighting the CHIA’s ability to adapt based on the user’s previous interactions.

## Discussion

### Principal Findings

To our knowledge, the CHIA is the first LLM-based conversational chatbot grounded in both MI and TTM to deliver PrEP and HIV prevention counseling and facilitate linkage to PrEP care. This approach represents a significant advancement over traditional chatbots based on natural language processing or machine learning, which rely on prewritten scripts or rule-based logic [54]. The CHIA leverages dynamic conversational AI to offer personalized, contextually relevant responses tailored to users’ readiness for behavior change. Internal testing demonstrated that the CHIA performs well across both English and Spanish, with relatively high scores in accuracy, trustworthiness, up-to-dateness, conciseness, and most metrics that assessed alignment with the MI spirit. These findings suggest that CHIA has the potential to deliver scalable, high-quality MI-aligned counseling, based on its technical design features such as 24/7 availability and low marginal cost per user. However, we emphasize that scalability was not evaluated in this internal testing and remains a theoretical advantage; the CHIA was assessed only for feasibility and usability in a controlled setting. A full assessment of feasibility, cost-effectiveness, cultural sensitivity, and barriers to real-world scalability will be a focus of the planned randomized controlled trial and subsequent implementation studies.

Although the CHIA received high scores in accuracy and many other assessment metrics, the findings from our internal testing also highlight areas for refinement to further enhance its MI metric-based performance. Beyond accuracy, mimicking human-led MI sessions is critical for improving engagement and behavior change [17]. AI-based chatbots must be trained not only to retrieve and deliver accurate health information but also to apply MI-consistent behaviors, such as open-ended questioning and reflective listening, to foster user motivation and self-efficacy [17]. While the CHIA is capable of providing MI-aligned counseling, there is room to strengthen its ability to engage users more deeply through improved use of MI-consistent responses. MI-based metrics received lower scores, especially in the Spanish version, and the CHIA may benefit from targeted improvements through additional prompt engineering and preference fine-tuning. A Spanish-fluent member of our study team will assist with prompt engineering and optimizing the CHIA for Spanish speakers. The Spanish language responses were generally strong; however, improving cultural and linguistic alignment through more intentional prompt design could strengthen its ability to deliver MI-based counseling even further. A key limitation of our internal testing was the absence of double scoring; future rounds of testing prior to the pilot phase will include 2 independent raters and a consensus process.

To improve the CHIA’s responsiveness and alignment with MI, we plan to integrate reinforcement learning into its training process [55]. Reinforcement learning approaches further optimize chatbot responses over time by integrating user feedback and adapting to real-world interactions [55]. Specifically, we will implement the Q-Star algorithm [56], a Q-learning–based approach designed to iteratively optimize performance based on feedback from MI-based metric assessments [57,58]. Q-Star is compatible with the AutoGen framework and will allow us to incorporate a dedicated Q-Agent that learns from evaluation data and adjusts the CHIA’s decision-making over time [56]. By continuously refining responses based on user interactions and alignment with MI, this approach offers an adaptive pathway to improve the CHIA’s accuracy, engagement, and overall quality of counseling.

The CHIA’s core functions (ie, referral for clinical services and HIV risk assessment) performed well during internal testing, successfully delivering relevant information and supporting user needs. However, several areas for improvement were identified to enhance functionality and user experience. For example, the “search for providers” function could be expanded to allow users to access more detailed information about clinics in which they express interest, including services offered, hours, and contact details. The HIV risk assessment function may benefit from an override option that allows users to opt out when they indicate they do not wish to be assessed, thereby supporting autonomy and comfort. Additionally, the “call for real-person support” function could be strengthened by asking more specific questions and capturing details—such as the user’s current concern, emotional state, or preferred mode of contact—that would better prepare research assistants to offer timely, personalized support. This function could be triggered when the user expresses uncertainty, distress, or repeatedly requests help, signaling the need for human follow-up. Overall, these targeted improvements represent feasible next steps to further tailor the CHIA’s functions to support users’ preferences and needs.

Although the CHIA demonstrated decent retrieval efficacy during internal testing, there is room for improvement. While the Spanish responses received higher mean scores in accuracy compared to the English responses, it is important to note that the Spanish version was assessed at a later stage in which the coding team had resolved several multilingual processing issues and patched the retrieval function, which likely resulted in enhanced accuracy. We plan to further expand our retrieval strategy across both languages by incorporating multiple related questions into each embedded response to enhance the relevance and completeness of retrieved information. Additionally, we will restructure the embedded dataset into smaller, topic-specific subsets to improve both retrieval speed and accuracy. To minimize hallucinations and maintain response quality, we will continuously monitor advancements in RAG techniques and integrate improvements as appropriate. Finally, we will track the performance of various LLMs and remain open to adopting alternative models that may better align with the CHIA’s counseling objectives and technical needs.

While engagement with chatbots for counseling typically involves short sessions of 3-10 minutes [59,60], insufficient engagement with the CHIA could hinder its effectiveness in real-world settings [61]. To strengthen engagement in the planned iterative refinement phase, the CHIA will offer brief, MI-structured exchanges (engage-focus-evoke-plan) with proactive reengagement and personalization. Sessions will be concise yet purposeful, while teachability preserves consented context (identity token, risk or concerns, prior plan) so return visits feel continuous and tailored. Between sessions, the CHIA will deliver targeted reminders and follow-up prompts, maintain an encouraging, human-like tone (with judicious emoji where appropriate), and surface just-in-time content aligned with user goals. A streamlined interface (clear navigation, minimal friction) will reduce drop-offs, and on-demand human support will be available for safety, complexity, or user preference. These approaches have the potential to refine the CHIA’s performance, improve satisfaction, and support repeat use in real-world settings.

### Conclusions

In summary, the internal testing of CHIA demonstrated promising performance in delivering MI-based counseling for HIV and PrEP education in English and Spanish. The chatbot performed well in key areas, including accuracy, trustworthiness, up-to-dateness, conciseness, and overall alignment with the MI spirit. However, targeted refinements are needed, particularly in enhancing MI alignment in Spanish responses to promote the CHIA’s acceptability among Spanish-speaking populations. Planned enhancements, such as improved retrieval strategies, reinforcement learning through Q-Star, and iterative prototype refinement based on user feedback, will further strengthen the CHIA’s ability to deliver responsive and user-centered support. The assessment of real-world effectiveness of its potential for supporting PrEP uptake is planned for a subsequent phase, during which stigma and discrimination outcomes will be explicitly measured alongside counseling effectiveness. These efforts position the CHIA as a potentially scalable and adaptable tool for promoting HIV prevention, supporting PrEP uptake, and offering valuable insight into the application of conversational AI in health interventions.

## Supplementary material

10.2196/79671 Multimedia Appendix 1 Operational rating scale for Chatbot for HIV Prevention and Action’s (CHIA) multilingual evaluation.

10.2196/79671 Multimedia Appendix 2 Researcher prompts, Chatbot for HIV Prevention and Action’s (CHIA) responses, and multilingual evaluation metric scores.

## Abbreviations

AI: artificial intelligence
AI: Artificial intelligence
AWS: Amazon Web Services
CHIA: Chatbot for HIV Prevention and Action
HIPAA: Health Insurance Portability and Accountability Act
LLM: large language model
MI: motivational interviewing
MSM: Men who have sex with men
PrEP: preexposure prophylaxis
RAG: retrieval-augmented generation
TTM: transtheoretical model
